# Augmented Reality of Anatomical Structures of the Maxillomandibular Complex for Learning Dental Radiographic Techniques

**DOI:** 10.1002/jdd.70112

**Published:** 2025-12-07

**Authors:** Maitê Michel Piazza Willig, Mariana Boessio Vizzotto, Henrique Timm Vieira, Priscila Fernanda da Silveira Tiecher, Thiago de Oliveira Gamba, Nádia Assein Arús, Luis Ernesto Arriola‐Guillén, Heraldo Luís Dias da Silveira

**Affiliations:** ^1^ Surgery and Orthopedics Department Federal University of Rio Grande do Sul Porto Alegre Brazil; ^2^ Federal University of Pelotas Rio Grande do Sul Brazil; ^3^ Division of Orthodontics School of Dentistry Universidad Científica del Sur Lima Peru

**Keywords:** augmented reality, dental student, learning

## Abstract

**Introduction:**

Augmented reality (AR) enables interactive learning in real‐world environments. In dental radiography education, AR offers a promising tool to enhance students' clinical skills by allowing them to simulate and practice procedures before engaging with actual patients. Repeated practice in an AR environment can reinforce theoretical knowledge and improve practical competence. Understanding dental anatomy and surrounding bone structures is essential for achieving accurate radiographic techniques, and 3D reconstructions from cone‐beam computed tomography (CBCT) aid this learning.

**Objective:**

To develop and evaluate an AR application designed to support the learning of periapical radiographic techniques.

**Methods:**

An AR application was developed using Digital Imaging and Communication in Medicine (DICOM) images, which were converted into Standard Tessellation Language (STL) format. Meshmixer software was employed to construct a three‐dimensional patient model, which was then used to create visual simulations of periapical radiographic projections based on established literature. The resulting images were integrated into the M3DMIX AR platform for visualization. Usability was assessed by 44 undergraduate dental students using a questionnaire based on the System Usability Scale (SUS).

**Results:**

The AR application achieved a mean usability score of 89.26 on the SUS, indicating a high level of user satisfaction. Students reported that the AR tool positively influenced their learning experience and enhanced their understanding of radiographic techniques.

**Conclusion:**

The AR application developed in this study demonstrated strong potential as an effective educational tool for teaching periapical radiographic techniques. Its high usability rating suggests that AR can significantly contribute to improving dental radiography education.

## 1 Introduction

Augmented reality (AR) and virtual reality (VR) are immersive technologies that reshape user interaction with digital content, yet they differ fundamentally in their relationship to the physical environment. AR enhances the real world by overlaying digital information—such as images, data, or 3D objects—onto the user's physical surroundings. Delivered through devices like smartphones, tablets, or AR glasses, AR maintains user awareness of the real environment while enabling interaction with virtual elements anchored in real space. In contrast, VR offers a fully immersive, computer‐generated environment that replaces physical reality. Using head‐mounted displays and motion tracking, VR serves sensory input from the real world, allowing users to navigate and interact within a virtual space [1].

VR technology is evolving rapidly, with new systems and applications emerging almost daily. Among the various technologies being integrated into education, AR is currently garnering particular attention [2]. The teaching of anatomy has traditionally relied on methods such as human cadaveric dissection and textbook‐based instruction. However, with advancements in technology, a variety of innovative learning techniques—such as VR simulations, 3D modeling, and interactive software—have emerged to complement or even replace traditional approaches [3, 4]. A systematic review evaluated the integration of VR and AR technologies in undergraduate and postgraduate education programs that include anatomy as a component of the curriculum. The review highlighted recent advancements in immersive technologies and assessed their effectiveness in enhancing anatomical understanding and learner engagement [5].

Previous studies have demonstrated encouraging results in the integration of VR tools into dental education. The interactive nature of VR enhances the learning process by providing realistic instruction for patient treatment, both through preclinical simulation labs and case‐based scenarios. In addition, VR tools contribute to the development of students' psychomotor skills [6, 7, 8]. Other authors have emphasized the importance of refining skills prior to practicing on patients, which increases the safety of the patients themselves, as students are better prepared to care for them [9].

The use of VR and AR has increased over the years, making them promising tools for enhancing the quality and accuracy of healthcare procedures [10]. The increased use of VR and AR tools highlights that new research [9] in these fields can enrich and improve the educational process in all areas of dentistry, including radiographic techniques and anatomy.

Periapical dental radiography plays a crucial role in accurate diagnosis and effective dental treatment planning [11]. Two primary techniques are used in periapical radiography: the paralleling technique and the bisecting angle technique. The paralleling technique involves placing the film parallel to the long axis of the tooth and directing the x‐ray beam perpendicular to both, thereby minimizing image distortion. In contrast, the bisecting angle technique positions the film as close to the tooth as possible, with the x‐ray beam directed perpendicular to an imaginary line that bisects the angle between the tooth and the film. This method is typically used when the paralleling technique is not feasible. However, it demands precise angulation, as incorrect positioning can result in image elongation or foreshortening [12].

Therefore, a comprehensive understanding of radiographic acquisition techniques, along with the ability to accurately interpret images, is essential in dental education [13]. In response to this need, the present study aimed to develop and evaluate the usability of an AR application designed to support the learning and application of periapical radiographic techniques.

## 2 Materials and Methods

### 2.1 Study Design

The present study followed a prospective observational design. The research was conducted at the Digital Image Processing Laboratory (LAPID) of the Faculty of Dentistry at the university.

### 2.2 Development

The AR application was developed using cone‐beam computed tomography (CBCT) scans obtained from the archives of a radiology center. The CBCT images were acquired with an Orthopantomograph OP 3D PRO system (KaVo Dental, Biberach an der Riß, Baden‐Württemberg, Germany), using an extended field‐of‐view (FOV) protocol measuring 13 × 15 cm and a voxel size of 0.32 mm. The study included two scans from a single patient, who had previously undergone CBCT imaging of the maxilla and mandible in both open‐ and closed‐mouth positions.

**FIGURE 1:**
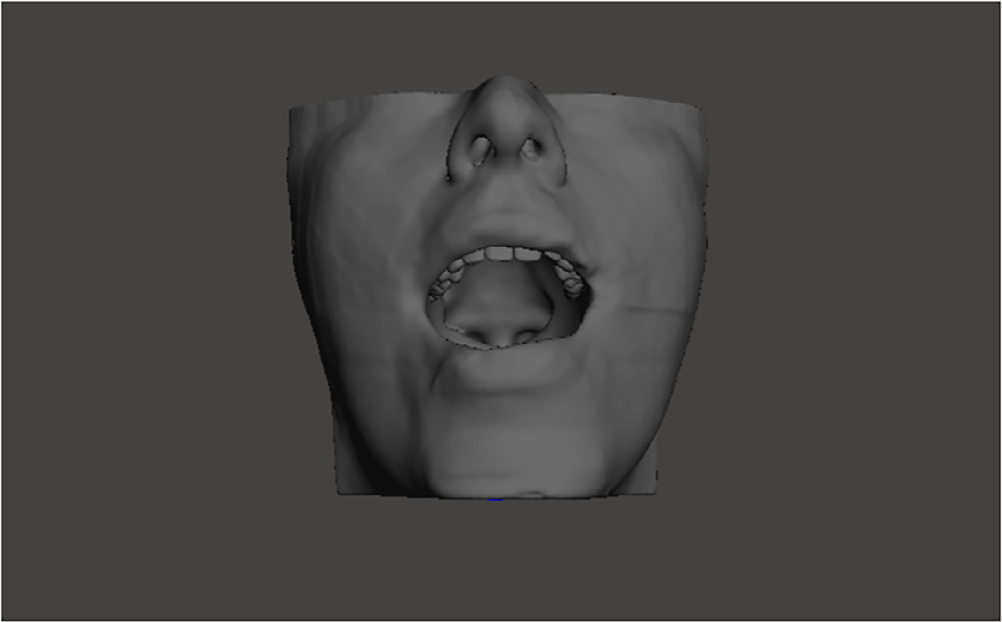
Standard Tessellation Language image in Meshmixer software.

Digital Imaging and Communications in Medicine (DICOM) images were converted to Standard Tessellation Language (STL) files using Diagnocat AI software (San Francisco, CA, USA). The STL file format represents the geometry of a three‐dimensional object by encoding its external surfaces as a mesh of interconnected triangular facets [14]. The STL files were imported into Meshmixer software (Autodesk, Mill Valley, CA, USA) to construct 3D models of the patient and to develop simulations for practicing periapical radiographic techniques, as illustrated in Figure 1. To replicate the X‐ray setup, additional 3D objects were modeled to represent the device, including the area and point of incidence, as well as a Size 2 phosphor plate.

**FIGURE 2:**
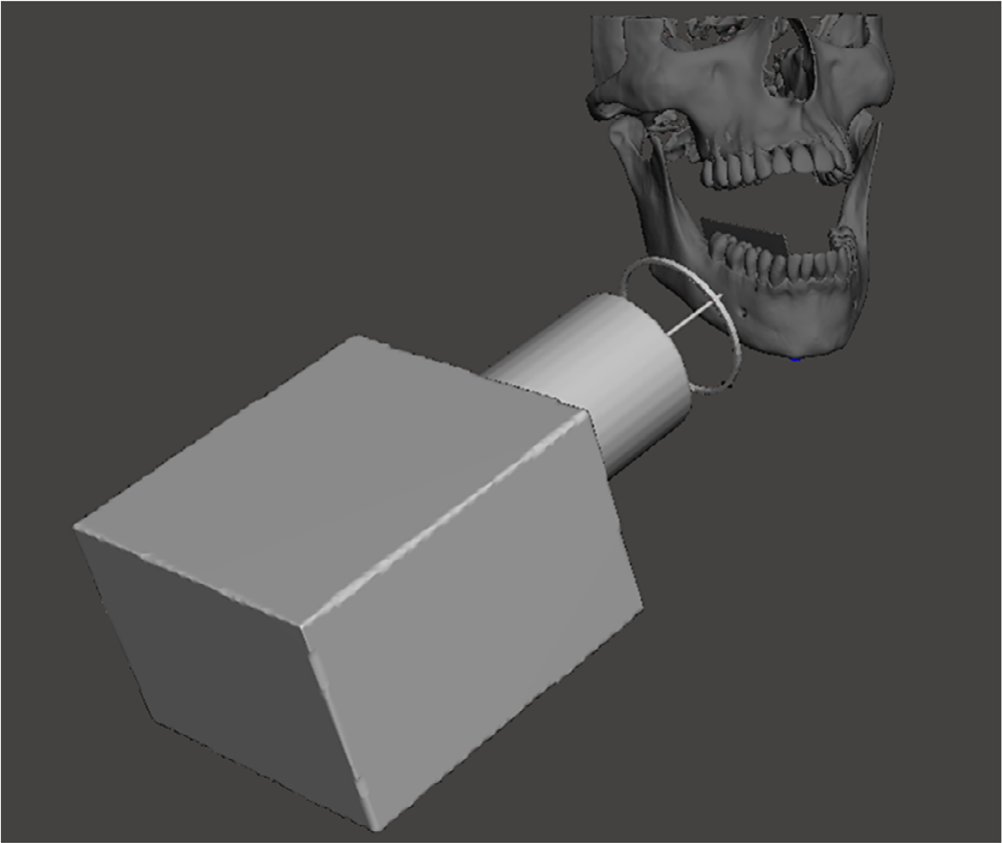
Radiographic technique during device development in Meshmixer.

Images demonstrating the appropriate positions for performing periapical radiographic techniques were developed based on the literature [12]. The angulation guidelines for projections followed the bisector technique, with specific projection configurations defined for each anatomical region: upper incisors, +45 to +55; lower incisors, −25 to −15; canines upper, +40 to +50; lower canines, −20 to −10; upper premolars, +30 to +45; lower premolars, −10 to −5; upper molars, +20 to +35; and lower molars, −5 to 0, as shown in Figure 2.

**FIGURE 3:**
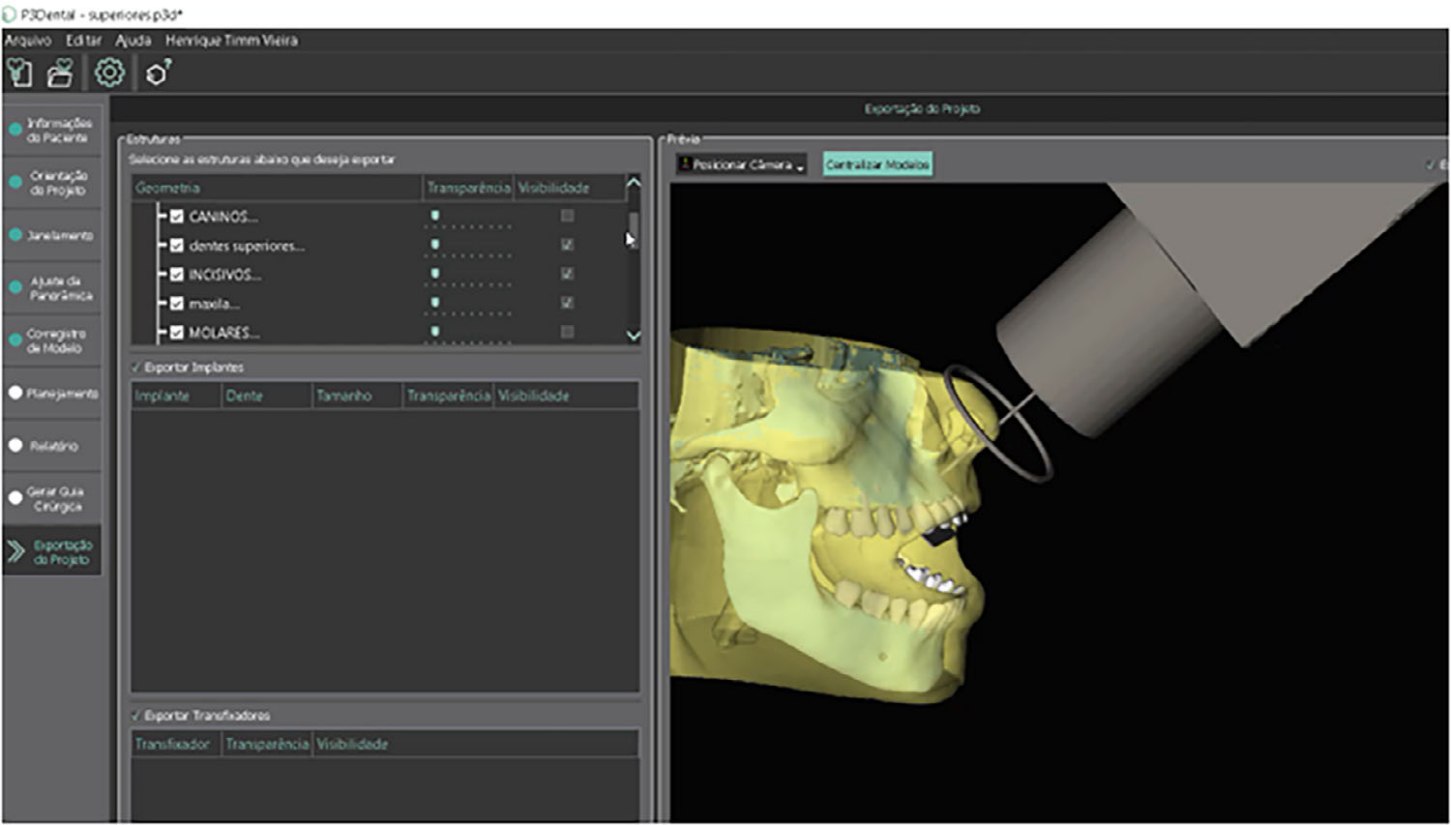
Projection creation in P3Dental.

The device images developed in Meshmixer were imported into P3Dental (P3DMed, Porto Alegre, Rio Grande do Sul, Brazil), a surgical planning software for implant dentistry, which was used to create visualizations of the projections of the maxillary and mandibular periapical radiographic techniques (Figure 3), to be visualized in AR using M3DMIX (P3DMed, Porto Alegre, Rio Grande do Sul, Brazil). M3DMIX generates a quick‐response code (QR code), printed in the shape of a cube, so that students can handle it from all angles and visualize all anatomical faces of the maxillomandibular complex and periapical radiographic techniques. This QR code is read using M3DMIX and used to visualize periapical radiographic techniques in AR.

### 2.3 Visualization of Images in AR

**FIGURE 4:**
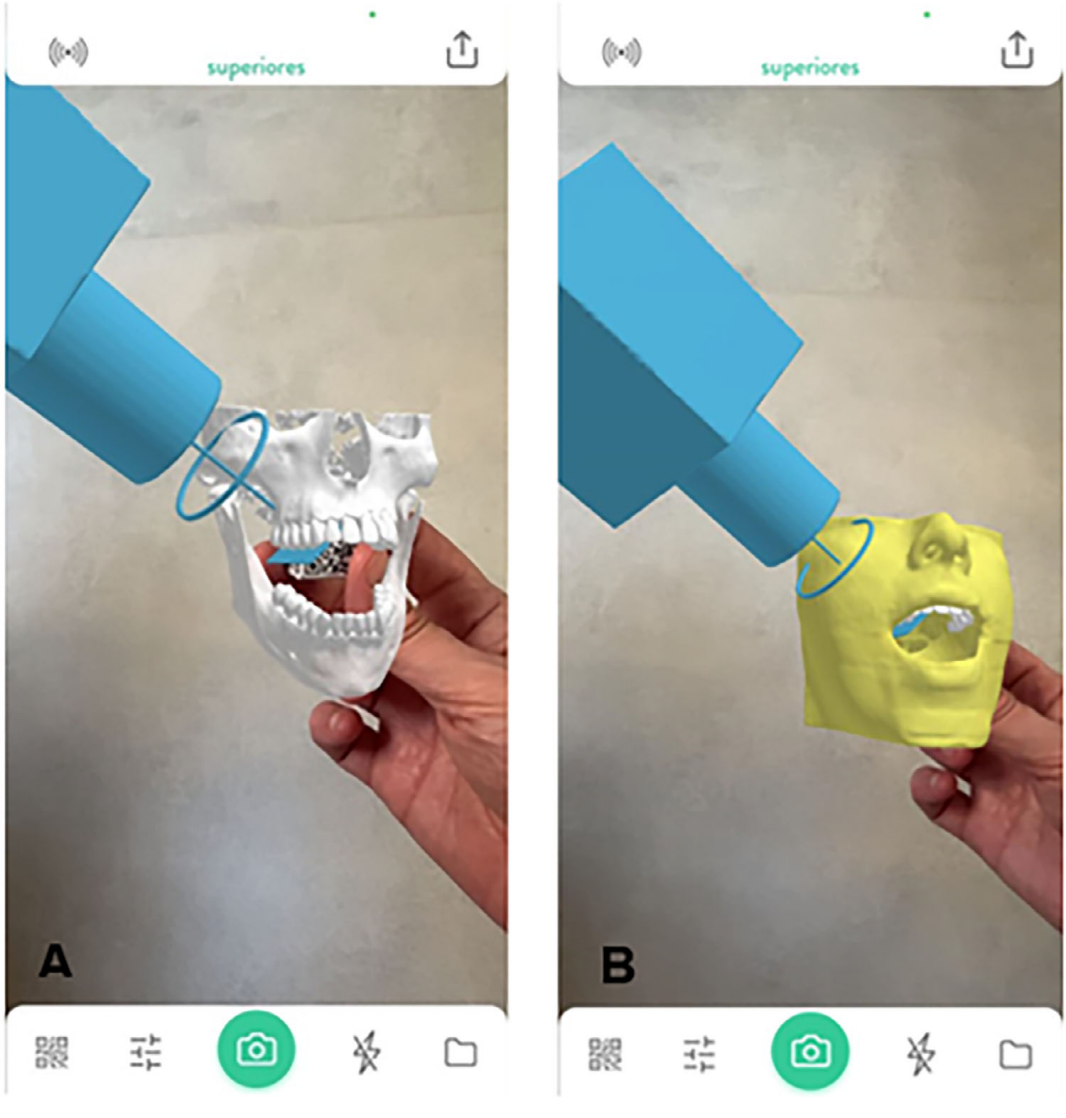
Screenshot of the AR visualization of a radiographic technique, with area and point of incidence.

The AR images can be viewed from any smartphone or tablet that has Android (Android Inc., Palo Alto, CA, USA), an operating system that operates on smartphones and tablets, or IOS (Apple Computer, Inc., Cupertino, CA, USA), Apple's mobile operating system developed for iPhone and iPad, whereby a QR code is read to obtain the AR image and periapical radiographic techniques, which can be viewed through M3DMix (Figure 4).

### 2.4 Device Usability Testing

To evaluate the usability of the device, all second‐year undergraduate dental students enrolled in the radiographic technique course—part of the dentistry curriculum at the university—were invited to participate in the study (*N *= 44). As the sample represents the entire population for that academic year, a priori power analysis was deemed unnecessary. The invitation was extended by a master's student (MSc) after the participants had received instruction on the bisector periapical radiographic technique.

Students accessed AR images via smartphones by scanning a QR code cube, which redirected them to the M3DMix application. Within this platform, they were able to visualize and interact with AR content designed to support learning in periapical radiographic techniques. The AR tool allowed students to view radiographic positioning from multiple perspectives, including the x‐ray tubehead, the intraoral plate, and various external angles. They could also manipulate virtual objects within the environment, enabling a deeper understanding of spatial relationships and positioning principles essential for accurate radiographic imaging. The test session lasted 50 min and was conducted in a silent room maintained at a constant temperature of 24°C, with a maximum of two participants per session. A Samsung A31 smartphone (Yeongtong District, Suwon, South Korea) was used, featuring a 6.4‐inch screen, 2400 × 1080 pixel resolution, a 48‐megapixel rear camera, 128 GB of internal storage, and 4 GB of RAM.

**FIGURE 5:**
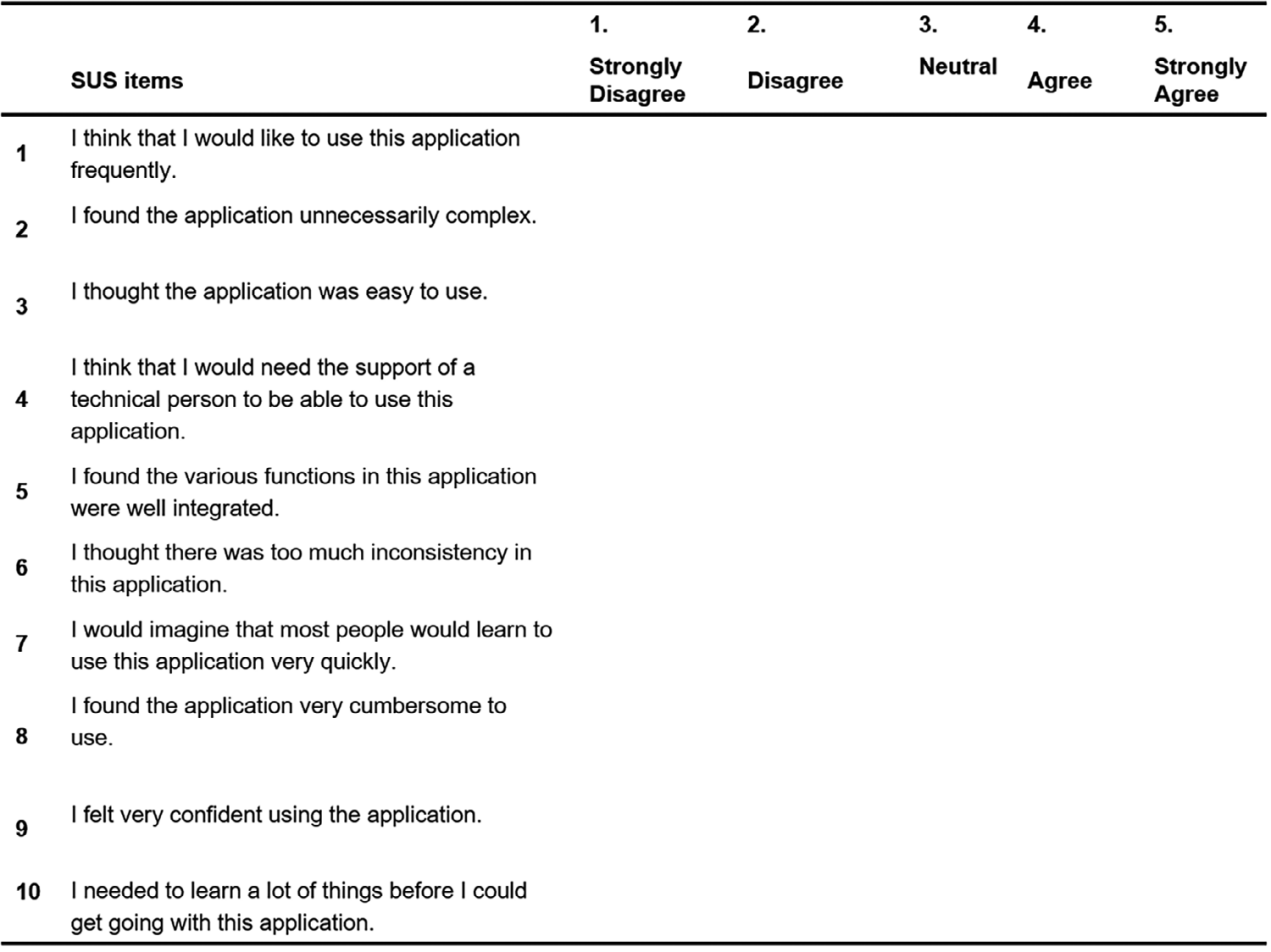
System Usability Scale (SUS) questionnaire.

To evaluate the effectiveness of the application as an auxiliary tool for learning periapical radiographic techniques, students were asked to complete a questionnaire based on the System Usability Scale (SUS) [15]. The SUS consists of 10 statements designed to assess the usability of a system, alternating between positive and negative phrasing (Figure 5).

The SUS questionnaire includes 10 items rated on a 5‐point Likert scale, where 1 indicates “*strongly disagree*,” and 5 indicates “*strongly agree*.” To calculate the SUS score, items 1, 3, 5, 7, and 9 are scored by subtracting 1 from the user response (i.e., score = response − 1), while items 2, 4, 6, 8, and 10 are scored by subtracting the response from 5 (i.e., score = 5 − response). The adjusted scores are summed and then multiplied by 2.5, resulting in a final SUS score ranging from 0 to 100, representing the overall usability of the system. Within a curved grading scale, a score of 68 is designated as the midpoint, functioning as a benchmark for distinguishing between below‐average and above‐average usability. Scores slightly above this threshold, such as 70, are typically interpreted as indicative of good usability, while scores exceeding 80 are classified as excellent [16].

The students completed the SUS questionnaire after interacting with the AR application on a mobile phone. Each question was formulated such that users could evaluate different aspects of usability and their experience with AR. Questionnaire responses were collected individually, without the presence of an evaluator, to prevent any potential influence on the results. In addition, to gather quantitative feedback, five follow‐up questions rated on a 5‐point Likert scale were included, focusing on participants' satisfaction and perceptions of the AR application's effectiveness in facilitating the learning of periapical radiographic techniques. The statements were as follows:

I felt more motivated to learn radiographic techniques using this AR mechanism.

I believe that the experience will be crucial for consolidating knowledge in my learning process.

I believe that the use of new technologies in the teaching environment has a positive impact on the learning process.

I would be willing to use this device again for educational purposes.

I consider this device tiring for learning radiographic techniques. I consider this AR mechanism somewhat tiring for learning radiographic techniques.

### 2.5 Statistical Analysis

Data were analyzed using descriptive statistics in Microsoft Excel (Microsoft Corp., Redmond, WA, USA).

## 3 Results

Of the 49 eligible students, 44 agreed to participate in the application usability test. All participants received standardized training on the device's operation before reading and signing the informed consent form. The overall SUS scale was 89.26, as seen in Table 1, which represents a high level of usability in relation to the effectiveness, efficiency, and satisfaction of the AR device.

**TABLE 1:** Descriptive and inferential statistics of SUS scores by gender.

Metric	Male	Female	Total
Sample size (%)	11 (25%)	33 (75%)	44 (100%)
Mean	87.50	89.85	89.26
Standard deviation	7.16	10.42	—
*p* value	—	—	0.413

Among the 44 participants, 25% were male, and 75% were female. A comparison of SUS scores between male and female participants is presented in Table 1. The mean SUS scores were comparable across genders, and statistical analysis using the Student's *t*‐test indicated no significant difference between the two groups (*p* = 0.413). Of the 44 participants, 42 (95.5%) agreed with the statement that they would like to use the device frequently, with 63.6% indicating strong agreement. In addition, 70.5% of participants agreed that the device was not complex to use. Only two participants (4.5%) responded neutrally regarding the complexity of the device.

Most of the students (97.7%) found the device easy to use, with only 2.3% feeling neutral regarding the ease of use; however, when asked about the need for technical support from a person to be able to use this device, 4.5% were neutral on this aspect, and 2.3% agreed. When asked if the device was difficult to use, 6.8% agreed (Table 2).

**TABLE 2:** Percentage of responses to the System Usability Scale (SUS) questionnaire.

	SUS item	Strongly disagree (%)	Disagree (%)	Neutral (%)	Agree (%)	Strongly agree (%)
1.	I think that I would like to use this application frequently.	—	2.3	2.3	31.8	63.6
2.	I found the application unnecessarily complex.	70.5	25	4.5	—	—
3.	I thought the application was easy to use.	—	—	2.3	31.8	65.9
4.	I think that I would need the support of a technical person to be able to use this application.	70.5	22.7	4.5	2.3	—
5.	I found the various functions in this application were well integrated.	—	—	—	27.3	72.7
6.	I thought there was too much inconsistency in this application.	72.7	20.5	4.5	2.3	—
7.	I would imagine that most people would learn to use this application very quickly.	—	4.5	4.5	36.4	54.5
8.	I found the application very cumbersome to use.	70.5	22.7	4.5	2.3	—
9.	I felt very confident using the application.	—	—	11.4	43.2	45.5
10.	I needed to learn a lot of things before I could get going with this application.	68.2	25	2.3	4.5	—

Furthermore, everyone agreed that the various functions of the device were well integrated, with a total agreement of 72.7%. Only 4.5% felt that there were many components to learn to use the AR device. In addition, 72.7% completely disagreed with the question of whether this system was inconsistent. More than half of the students (54.5%) strongly agreed that most people would learn to use this device quickly, while 36.4% agreed, 4.5% were neutral, and only 4.5% disagreed. Most students felt confident about using the AR device, while only five were neutral.

Regarding feedback on the learning experience, 86.4% of students reported feeling more motivated to learn radiographic techniques using the device, and the majority (95.5%) agreed that the experience was important for consolidating their knowledge during the learning process (Table 3). All the students agreed that the use of new technologies in the teaching environment had a positive impact on the learning process and were willing to use the AR device again for educational purposes, while 2.3% found the device to be tiring.

**TABLE 3:** Questionnaire on feedback regarding the learning experience.

Question	Strongly disagree (%)	Disagree (%)	Neutral (%)	Agree (%)	Strongly agree (%)
1. I felt more motivated to learn radiographic techniques using this AR mechanism.	0	0	6 (13.6%)	16 (36.4%)	22 (50%)
2. I believe that the experience will be crucial for consolidating knowledge in my learning process.	0	0	2 (4.5%)	11 (25%)	31 (70.5%)
3. I believe that the use of new technologies in the teaching environment has a positive impact on the learning process.	0	0	0	3 (6.8%)	41 (93.2%)
4. I would be willing to use this device again for educational purposes.	0	0	0	8 (18.2%)	36 (81.8%)
5. I consider this AR mechanism somewhat tiring for learning radiographic techniques.	37 (84.1%)	5 (11.4%)	1 (2.3%)	0	1 (2.3%)

## 4 Discussion

The increasing adoption of virtual learning tools in medical and dental education reflects a broader trend toward integrating technology‐enhanced learning strategies into conventional curricula [2, 3, 4]. Such tools aim to address challenges in traditional education, including limited hands‐on opportunities and variability in instructional quality. Busanello et al. [11] reported that students who utilized virtual learning platforms demonstrated significantly improved academic performance compared to those receiving only traditional instruction. These findings support the potential of AR and similar technologies to improve student engagement, comprehension, and skill acquisition. The current study evaluated the usability of an AR application designed to facilitate the learning of radiographic techniques in dentistry. The results highlight not only the device's functionality and user‐friendliness but also its potential to reinforce theoretical knowledge through immersive, interactive experiences. These outcomes contribute to a growing body of evidence underscoring the relevance and effectiveness of integrating emerging technologies, such as AR, into educational environments to enhance teaching and learning outcomes.

In the present study, dental students were selected to evaluate the usability of an AR application, aligning with findings from a previous systematic review [17]. That review noted that the majority of studies exploring AR in dental education focused on undergraduate learners and emphasized that AR serves as a valuable complement to traditional teaching methods. Despite its potential, the broader adoption of AR in dental education remains limited by certain challenges, including a lack of standardized protocols and insufficient usability testing of AR technologies [18, 19, 20]. Addressing this gap, the current study assessed the usability of an AR device and collected user feedback. Results indicated that all participating students believed the integration of new technologies enhances the educational experience, and notably, 86.4% reported feeling more motivated to learn radiographic techniques through the use of AR.

A previous study [21] analyzing students' learning of root canal anatomy using CBCT scans, periapical radiographs, and VR/AR reported enhanced understanding of dental anatomy and improved knowledge retention with the use of immersive technologies. These findings are consistent with the results of the present study, in which students responded positively to learning periapical radiographic techniques through AR. The AR experience was not only well‐received but also appeared to support the consolidation of theoretical and practical knowledge, as evidenced by a 95.5% acceptance rate among participants. This high level of engagement suggests that AR can be an effective supplementary tool in dental education, enhancing both comprehension and student satisfaction.

The integration of digital learning objects alongside practical exercises has been shown to enhance students' understanding of key factors influencing radiographic imaging, including the positioning of the x‐ray device, the object being imaged, and its spatial orientation [22, 23]. These tools support the development of spatial reasoning and technical accuracy, which are essential competencies in dental radiography. In recent years, electronic learning (e‐learning) has gained traction as a complementary component of dental curricula, augmenting traditional teaching methods with flexible, interactive resources. Systematic reviews reinforce this trend, indicating that the incorporation of digital media into dental education is associated with positive learning outcomes and increased student engagement [17, 19].

Gu and Lee [18] developed an AR‐based simulator that enabled students to acquire essential skills related to radiographic imaging, thereby enhancing their understanding of oral anatomy through immersive, technology‐driven learning. Their findings indicated that students showed improved perception of angulations and spatial relationships in radiographic projections. Based on these outcomes, the authors emphasized the growing necessity for AR integration in dental radiography education—an assertion that aligns with the relevance of the AR device evaluated in the present study.

In parallel, Reymus et al. [21] highlighted the need for ongoing research into the comparative effectiveness of virtual versus traditional instructional methods. Addressing this call, a future study is proposed in which students are divided into two groups: one receiving traditional instruction supplemented with VR/AR technology, and the other following a purely conventional curriculum. This comparative design would allow for a rigorous assessment of how AR, when combined with traditional teaching methods, influences students' understanding and clinical performance in radiographic techniques. Such research could provide valuable evidence on the pedagogical value of immersive learning tools in dental education.

A similar research model comparing traditional teaching with technology‐enhanced methods was employed in a previous study [13], which evaluated student learning outcomes through standardized testing. The findings demonstrated that computer‐assisted learning surpassed conventional instruction in terms of knowledge acquisition and retention. While the present study did not directly compare instructional methods, it lays the groundwork for future research investigating the relative effectiveness of traditional versus AR‐assisted learning in dental radiography education. However, the validity of using standardized tests as the sole measure of knowledge—particularly in healthcare education—has been questioned [24]. Knowledge assessments may not fully capture students' clinical reasoning, practical skills, or long‐term retention, emphasizing the need for more comprehensive evaluation frameworks.

Several studies [25, 26, 27] have also underscored the necessity for further research into the application of AR in dentistry. Key areas for future investigation include its impact on educational outcomes, day‐to‐day clinical performance, the implementation of randomized controlled trials, and the creation of standardized AR technologies to support broader adoption. Addressing these gaps will be essential for validating the pedagogical and clinical value of AR in dental education. In our study, the overall SUS score was 89.26. Given that scores above 80 are generally considered excellent [16], this result suggests that the product is both promising and well‐received by users. We aim to build on these findings through continued product development and more comprehensive evaluations in future research.

The AR application developed in this study allowed students to visualize radiographic techniques directly on a smartphone, significantly enhancing the accessibility and convenience of learning. The app facilitated the visualization of critical components such as soft tissue structures, the area of incidence, and radiographic projection points, which are fundamental to understanding dental radiographic techniques. This level of accessibility may reduce the time required for instruction, as students can review the material independently and repeatedly at their own pace. These findings are consistent with a previous study [26], which emphasized the value of digital tools in providing flexible, on‐demand access to educational content.

This advantage is further supported by Jasinevicius et al. [6], who found that students learning cavity preparation through conventional instruction required five times more faculty instructional time than those who used digital learning tools. Importantly, there were no statistically significant differences in the practical outcomes between the two groups. These findings suggest that digital methods—such as AR—can offer comparable educational effectiveness while optimizing instructional efficiency, reinforcing the potential of such technologies to enhance dental education.

Uruthiralingam and Rea [5] emphasized that as technology advances, new and innovative approaches to learning anatomy have emerged, particularly through the development of VR and AR technologies. These tools have been increasingly implemented in both undergraduate and postgraduate programs that include anatomy as part of the curriculum. This trend underscores the relevance of the AR device developed in the present study for integration into undergraduate dental courses, specifically for teaching radiographic techniques. The potential for inclusion in future curricula is further supported by the present study's findings, in which students unanimously reported that the AR tool had a positive impact on their learning experience and expressed a strong willingness to use it.

Moreover, research has shown that AR and VR technologies stimulate active engagement and deeper learning—benefits that align with the preferences and needs of Generation Z (those born between 1995 and 2010), who currently make up the majority of undergraduate students. This generation is characterized by a high degree of technological fluency, which influences how they access, process, and retain information. Prior studies have noted that Gen Z students often struggle with concentration and face challenges such as information overload, rendering traditional lecture‐based teaching methods less effective [28, 29]. In this context, the AR device developed in the current study represents a valuable and complementary educational tool tailored to the learning preferences of today's students, offering a more interactive and engaging alternative to conventional methods.

The students who participated in the present study responded positively to the questionnaire, expressing a desire to use the AR device frequently. They found the device intuitive and user‐friendly, and reported that its various functions were well integrated. This, in turn, enhanced their motivation to learn radiographic techniques through its use. These results align with previous studies [30, 31, 32], suggesting that visually rich and interactive learning tools can enhance students' ability to assimilate and retain information, thereby contributing to a more effective and engaging educational experience.

We present an AR application developed to enhance the understanding of dental anatomy and adjacent bone structures—key elements for mastering accurate radiographic techniques. This pilot study explores the effectiveness of AR as a tool for dental education. In future work, we plan to integrate AR with artificial intelligence (AI) to further advance the learning experience. This combined system will allow students to visualize proper radiographic positioning alongside the resulting image. In cases of incorrect technique, the AI will offer real‐time corrective feedback by suggesting appropriate equipment positioning and displaying the expected radiographic outcome.

## 5 Conclusion

The findings of this study demonstrate that students perceived the integration of new technologies into the educational environment as having a positive impact on the learning process. Specifically, the AR application developed for this study showed strong potential as a teaching aid for undergraduate dentistry students, achieving a high usability index in the context of dental radiography instruction.

## Funding

The authors have nothing to report.

## Ethics Statement

The study was approved by the ethics and research committee of the university under approval no. 6163411.

## Consent

The study participants read and signed the informed consent form.

## Conflicts of Interest

The authors declare no conflicts of interest.
